# Intronic branchpoint-to-acceptor variants underlying inborn errors of immunity

**DOI:** 10.70962/jhi.20250041

**Published:** 2025-07-17

**Authors:** Najiba Alioua, Nathalie Lambert, Mathilde Puel, Sylvain Hanein, Paul Bastard, Mathieu Fusaro, Marie Jaffray, Bernardita Medel, Lydia Khellaf, Yoann Seeleuthner, Mélodie Perin, Corinne Jacques, Marlène Pasquet, Laura Olivier, Fernando Sepulveda, Tom Le Voyer, Aurélie Cobat, Patrick Nitschké, Lionel Galicier, Nicolas Schleinitz, Eric Oksenhendler, Marion Malphettes, Bénédicte Neven, Despina Moshous, Felipe Suarez, Claire Fieschi, Jean-Laurent Casanova, Geneviève de Saint Basile, Guillaume Dorval, Capucine Picard, Jacinta Bustamante, Peng Zhang, Jérémie Rosain

**Affiliations:** 1 Study Center for Primary Immunodeficiencies, Necker Hospital for Sick Children, Assistance Publique Hôpitaux de Paris (AP-HP), Paris, France, EU; 2 Bioinformatic Platform, Institute of Genetic Diseases, Inserm U1163, Imagine, University of Paris Cité and Necker Federative Research Structure, Paris, France, EU; 3 Pediatric Immunology-Hematology and Rheumatology Unit, Necker Hospital for Sick Children, AP-HP, Paris, France, EU; 4 Imagine Institute, Inserm U1163, University of Paris Cité, Paris, France, EU; 5 Laboratory of Human Genetics of Infectious Diseases, Necker Branch, Inserm U1163, Necker Hospital for Sick Children, Paris, France, EU; 6 St. Giles Laboratory of Human Genetics of Infectious Diseases, Rockefeller Branch, Rockefeller University, New York, USA; 7 Immunology Department Laboratory, Referral Medical Biology Laboratory, Institut Fédératif de Biologie, Toulouse University Hospital Center, France, EU; 8 INFINITy, Toulouse Institute for Infectious and Inflammatory Diseases, Inserm U1291, CNRS U5051, University Toulouse III, Toulouse, France, EU; 9 Department of Pediatrics, Dieppe Hospital, Dieppe, France, EU; 10 Laboratory of Molecular Basis of Altered Immune Homeostasis, Imagine Institute, Inserm U1163, University of Paris Cité, Paris, France, EU; 11 Department of Pediatric Hematology and Immunology, Children’s Hospital, University Hospital, Toulouse, France, EU; 12 Immunopathology Department, Saint-Louis Hospital, AP-HP, University of Paris Cité, Paris, France, EU; 13 Department of Immunology, Timone Hospital, Assistance Publique des Hôpitaux de Marseille, Marseille Immunopole, Marseille, France, EU; 14 Department of Clinical Hematology, Necker Hospital for Sick Children, AP-HP, Paris, France, EU; 15 Department of Pediatrics, Necker Hospital for Sick Children, AP-HP, Paris, France, EU; 16 Howard Hughes Medical Institute, New York, NY, USA; 17 Laboratory of Hereditary Kidney Diseases, Imagine Institute, Inserm U1163, University of Paris Cité, Paris, France, EU; 18 Laboratory of Genomic Medicine for Rare Diseases, Necker Hospital for Sick Children, AP-HP, Paris, France, EU; 19 Laboratory of Lymphocyte Activation and Susceptibility to EBV Infection, Inserm U1163, Imagine Institute, Paris, France, EU; 20 Centre de Référence des Déficits Immunitaires Héréditaires (CEREDIH), Necker Hospital for Sick Children, AP-HP, Paris, France, EU

**Keywords:** Inborn error of immunity, genetics, intronic variants, branchpoint, pathogenicity

## Abstract

Clinical laboratories searching for pathogenic variants focus mostly on the protein-coding region and corresponding essential splicing sites. Screening for variants in intronic regions requires dedicated bioinformatics tools and detailed experimental studies to confirm deleteriousness and pathogenicity. We report intronic variants in a cohort of eight patients from seven kindreds with unexplained inborn errors of immunity (IEI). Using ad hoc bioinformatics tools, we identified seven kindreds carrying three branchpoint variants at three loci (*BTK*, *SH2D1A*, and *WAS*) and four AG-gain acceptor site variants at another three loci (*DOCK8*, *NFKB1*, *STXBP2*, and *UNC13D*). The variants were located between positions −9 and −49 relative to the wild-type acceptor site. The deleteriousness and, thus, pathogenicity of these variants were confirmed by exon-captured transcriptome studies and flow cytometry analyses of protein production or function. Our findings indicate that intronic variants should be systematically screened and investigated, even in clinical laboratory settings.

## INTRODUCTION

Genomic medicine is becoming more widely available worldwide, including for patients with suspected inborn errors of immunity (IEI) (1-13). Genetic diagnosis is essential for IEI to end the diagnostic odyssey and make it possible to initiate targeted and non-targeted therapies or prophylaxis, and genetic counseling. However, it has been estimated that no more than 40% of patients with suspected IEI receive a genetic diagnosis (9). Clinical laboratories focus their search for germline pathogenic variants on single-nucleotide variants (SNV), indels, and structural variants affecting the coding sequence (CDS) and essential splicing sites (1). The identification of variants in intronic regions would increase diagnosis rates for patients with IEI (14-16). Such variants can have pathogenic effects via various biological mechanisms. In particular, variants may disrupt intronic branchpoints (BP) (17) or lead to the gain of AG acceptor nucleotides between BP and canonical splice acceptor sites (18). Intronic branchpoint-to-acceptor variants are the most proximal, as 88% of BP variants are located between positions −40 and −15 relative to the canonical acceptor site (17). Such variants can, therefore, be covered even by high-throughput sequencing (HTS) approaches capturing exons, such as panel or whole-exome sequencing (WES). However, screening for such intronic variants requires dedicated bioinformatics tools, and several such tools have recently been developed (17-20). Additional wet-laboratory studies are also required to confirm the pathogenicity of the variants identified (14,21), which can be challenging in clinical laboratory settings.

## RESULTS

### Seven unrelated kindreds with IEI

We retrospectively report eight patients in whom we have ultimately identified BP and AG-gain variants in genes of IEI. The patients were initially referred to our clinical laboratory (Study Center for Primary Immunodeficiencies, Paris, France) based on their clinical phenotype and laboratory test results during basic immunological investigations. The eight patients (P1 to P8) were from seven unrelated kindreds (A to G). The case reports are described in detail Table S1 and the Supplementary Material. All the patients were living in France. The patients had a mean age of 29 years (range: 8-58 years); seven patients were male and one was female. One patient (P5) was born to consanguineous parents. P1 from kindred A had a history of invasive bacterial disease and agammaglobulinemia. P2 from kindred B had a history of Epstein-Barr virus (EBV)-negative diffuse large B-cell lymphoma and hypogammaglobulinemia. P3 from kindred C had eczema and thrombocytopenia. P4 from kindred D had a history of bronchiectasis, cutaneous and genital human papillomavirus (HPV) infections, and combined immunodeficiency. P5 and P6 from kindreds E and F, respectively, had a history of hemophagocytic lymphohistiocytosis (HLH). P7 and his father, P8, from kindred G had a history of common variable immunodeficiency (CVID). All patients were screened for IEI by targeted HTS encompassing all genes for which inborn errors of immunity are known (22). However, an analysis of SNV, indels, and copy-number variants (CNV) within coding sequences and essential splice sites identified no candidate pathogenic variants. Due to their clinical and immunological phenotypes, these patients were subsequently and sporadically referred back to our laboratory.

### Identification of BP or AG-gain variants in the seven kindreds

**Figure 1 –:**
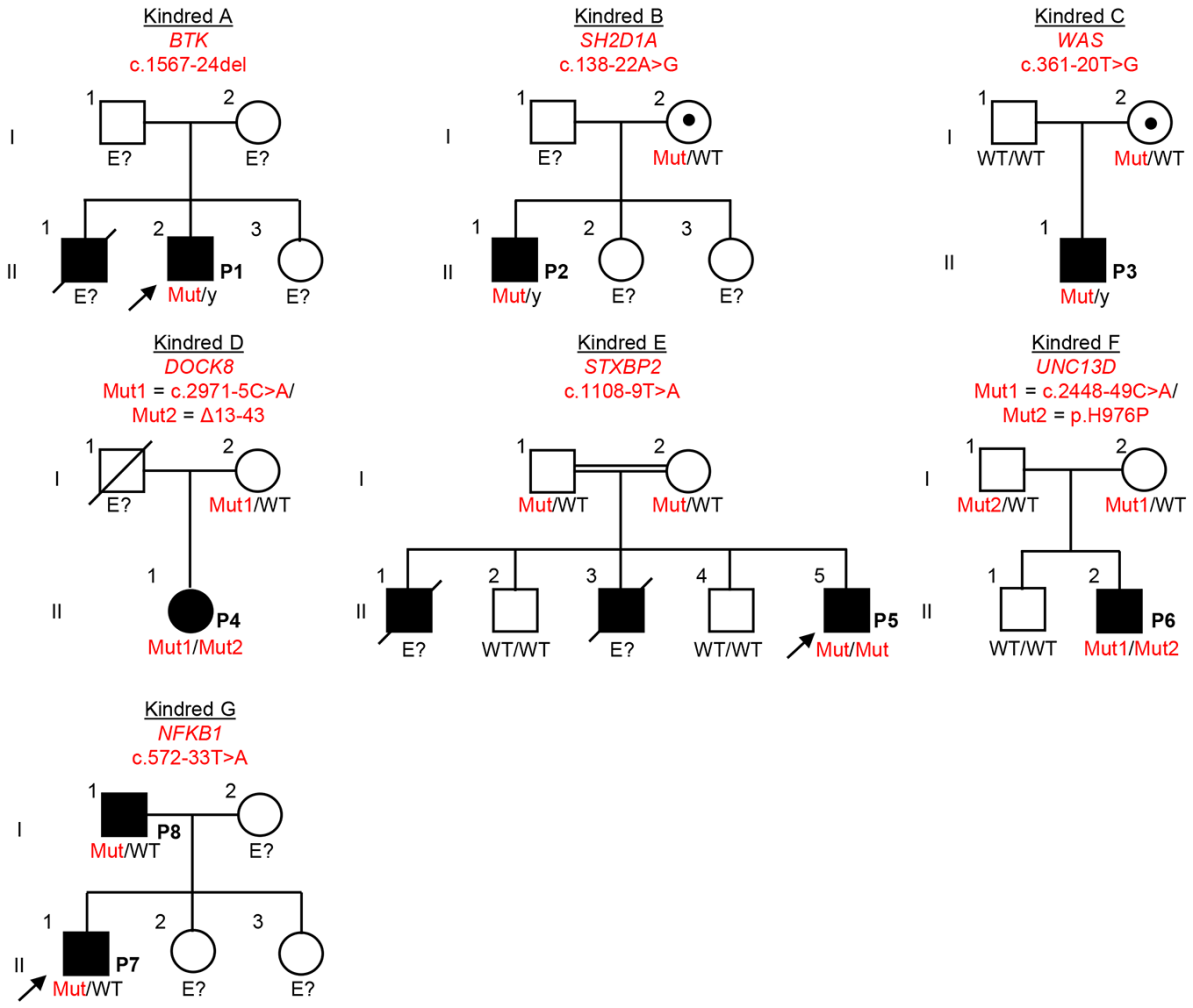
Pedigree of seven French kindreds with inborn errors of immunity. Male and female individuals are represented by squares and circles, respectively. Each generation is designated by a Roman numeral and each individual by an Arabic numeral. Individuals with immune dysregulation are shown as closed black symbols and the index case is indicated by an arrow. Individuals whose genetic status could not be tested are designated “E?”. Mut=mutated; WT=wild-type

Targeted HTS involves the capturing of exons with a mean coverage > 400X, resulting in partial coverage of the flanking intronic regions (10). We therefore reanalyzed HTS data of the patients, searching for candidate intronic variants, given the high level of suspicion for IEI in all kindreds. Variants were primarily screened using AGAIN (18), BPHunter (17), SpliceAI (19), and Pangolin (20), and were also subsequently analyzed with CADD v1.7 (23), phastCons (24), and phyloP (25). Interestingly, we identified candidate intronic variants in all kindreds: BP candidate variants at three loci (*BTK*, *SH2D1A*, and *WAS*) in kindreds A, B and C, respectively, and AG-gain candidate variants at another four loci (*DOCK8*, *STXBP2*, *UNC13D,* and *NFKB1)*, in kindreds D, E, F, and G, respectively (Table 1 and Figure 1). One variant was present in the homozygous state (*STXBP2*), three were hemizygous (*BTK, SH2D1A*, and *WAS*), two were heterozygous in *trans* with another heterozygous variant located in the CDS (a large deletion in *DOCK8* and a missense variant in *UNC13D*), and one was present in the heterozygous state (*NFKB1*) (Table 1 and Figure 1). In P4, a revertant in *DOCK8* c.2971-6C>G was evidenced on genomic DNA extracted from T-cell blasts (T-blasts). This revertant was flanking the germline intronic variant (c.2971-5C>A) (Figure S1). All the germline intronic branchpoint-to-acceptor variants were predicted to be deleterious by BPHunter (for BP variants) and AGAIN (for AG-gain variants), and all had SpliceAI and Pangolin scores above the cutoff of ∣0.2∣ (range=0.36-0.99) (Table 1). BP variants were scored higher than AG-gain variants by CADD, phyloP and phastCons (Table 1). All the variants identified were rare, and were absent from gnomAD v4.1 (26), BRAVO/TOPmed freeze 8 (27), UKBiobank (28), or All-of-Us (29) databases. None of these variants were reported in HGMD professional v2024.4 (30). Providing further support for their pathogenicity, these variants segregated with the clinical phenotype in the various kindreds (Figure 1). We, thus, identified rare and previously unknown candidate intronic variants in IEI genes with recessive or dominant inheritance in all the kindreds studied.

### Investigating the candidate intronic variants at the mRNA and protein levels

**Figure 2 –:**
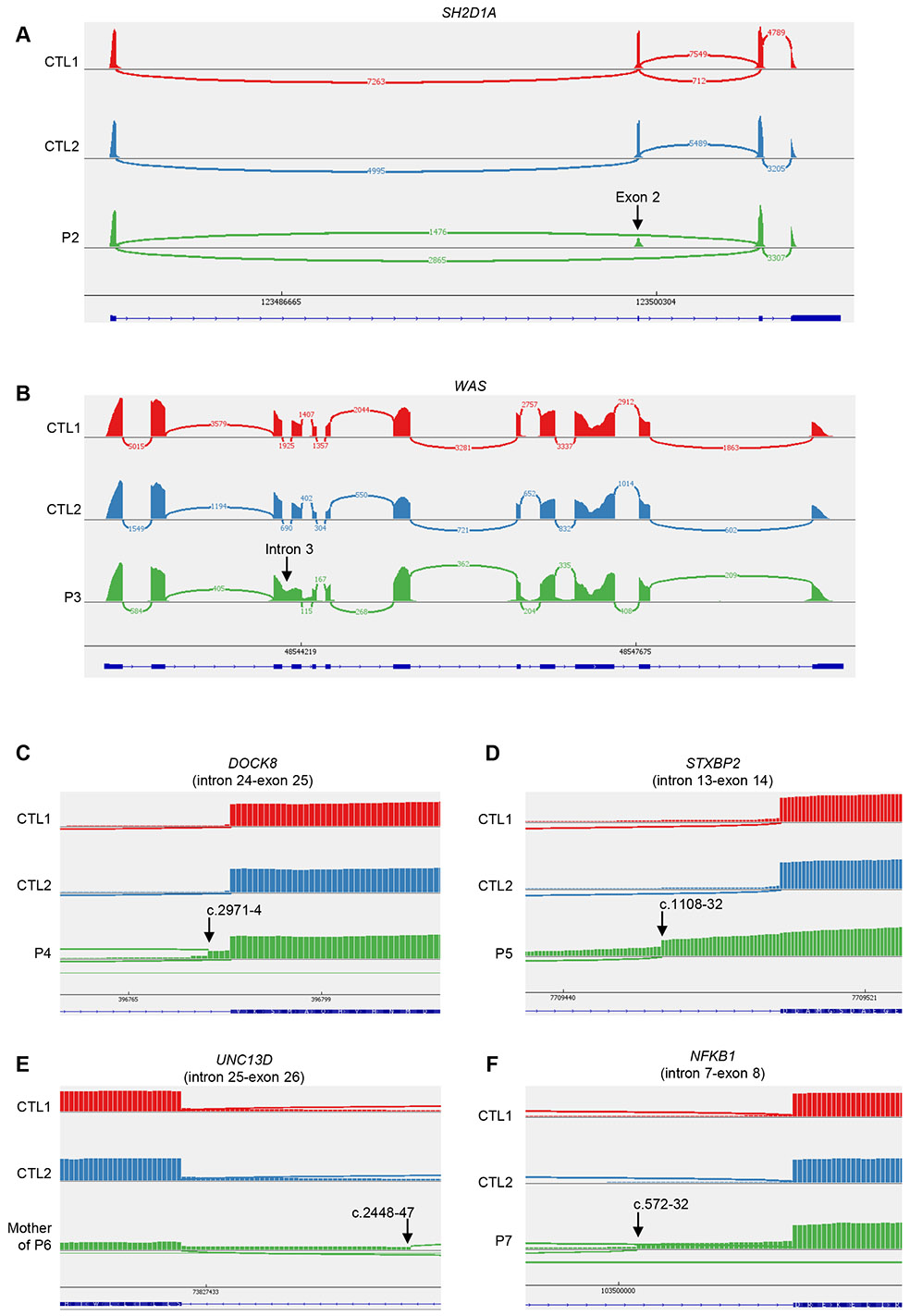
Sashimi view of RNA sequencing in three different kindreds with intronic variants affecting branchpoint sites or AG-gain variants located between the BP and the acceptor splice site. RNA-sequencing data from **(A)** T-blasts of P2 and two controls (CTL), **(B)** T-blasts of P3 and two controls (CTL), **(C)** T-blasts of P4 and two controls (CTLs), **(D)** T-blasts of P5 and two controls (CTLs), **(E)** PBMCs of the mother of P6 and two controls (CTLs), and **(F)** T-blasts of P7 and two controls (CTLs).

**Figure 3 –:**
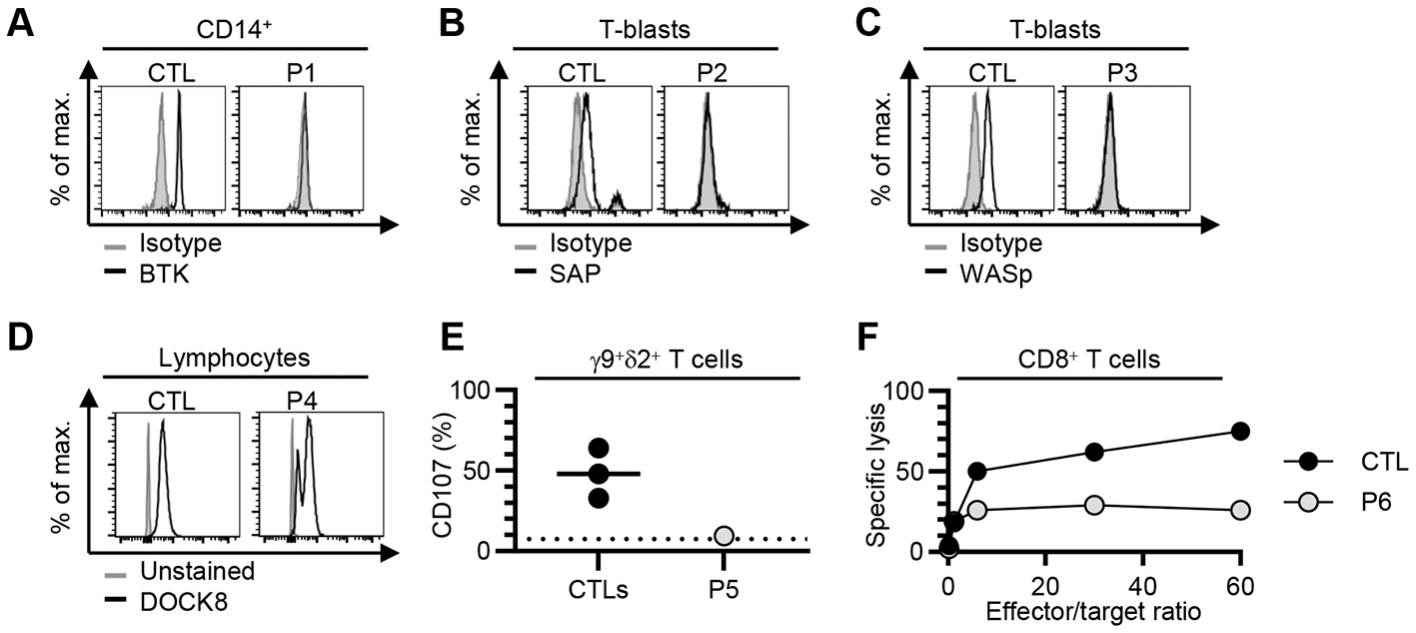
Intracellular protein expression or functional assays for the index cases from kindreds A, B, C, D, E and F. Flow cytometry study of **(A)** BTK gated on CD14^+^ cells from fresh whole blood from P1 and a control (CTL), **(B)** SAP in T-blasts from P2 and a CTL, **(C)** WASp in T-blasts from P3 and a CTL, and **(D)** DOCK8 in lymphocytes from the fresh whole-blood cells of P4. **(E)** Degranulation assay on Vγ9^+^Vδ2^+^ T cells stimulated with HMBPP in three controls (CTLs) and P5. **(F)** ^51^Cr-based cytotoxic activity of Fas-deficient L1210-3 target cells in CD8^+^ T cells from a control (CTL) or P6. The results are expressed as the percent specific lysis. The effector/target ratio is shown on the *x*-axis and was calculated from the number of CD8^+^ T cells, as determined by flow cytometry

We then studied the consequences of these variants for RNA splicing, protein production and/or function. We used peripheral blood cells from the patients or their siblings, or cells derived from blood cells. For RNA splicing, we used the exon-captured transcriptome of the peripheral blood mononuclear cells (PBMCs) or T-blasts from the patients or their siblings. We performed transcriptomic analyses in all kindreds except kindred A. Exon 2 skipping occurred in the T-blasts of P2, who was hemizygous for the BP variant c.138-22A>G of *SH2D1A* (Figure 2A). In T-blasts from P3, who was hemizygous for the BP variant c.361-20T>G of *WAS*, we observed a retention of intron 3 that was predicted to cause a frameshift (Figure 2B). In the T-blasts of P4, exons 13 to 43 were skipped in the *DOCK8* transcript, due to the large deletion (Figure S2A), and there was a predicted frameshift insertion of 4 bp (r.2970_2971insACAG, p.V991Gfs*11), probably due to the c.2971-5C>A variant (Figure 2C). There were also, normal remaining skipping of exon 24 to 25 (Figure 2C), likely due to the revertant. In the T-blasts of P5, who was homozygous for the AG-gain variant c.1108-9T>A of *STXBP2*, a new acceptor splice site was created at c.1108-32, leading to a 32 nt frameshift insertion (r.1107_1108ins32, p.D370Rfs*6) (Figure 2D). In PBMCs from the mother of P6, who was heterozygous for the c.2448-49C>A variant of *UNC13D*, we observed the creation of a new acceptor splice site at c.2448-47, leading to a 47 nucleotides insertion with a frameshift (r.2447_2448ins47, p.L817Hfs*18) (Figure 2E). In the T-blasts of P7, who is heterozygous for the AG-gain variant c.572-33T>A of *NFKB1*, a new acceptor splice site was created at c.572-32 leading to a 32 nucleotides insertion with a frameshift (r.571_572ins32, p.D191Efs*3) (Figure 2F), and exon 8 was skipped, also leading to a frameshift (Figure S2B). Furthermore, we observed impaired production of the following proteins: (i) BTK in monocytes from P1 (Figure 3A), (ii) SAP in T-blasts from P2 (Figure 3B), (iii) WAS in T-blasts from P3 (Figure 3C), (iv) DOCK8 in lymphocytes from P4 (Figure 3D). P4 had two peaks of DOCK8 protein levels of different intensities, confirming at protein level the reversion (31). In addition, degranulation and cytotoxic assays revealed an impairment of Vγ9^+^Vδ2^+^ T cells from P5 and CD8^+^ T cells from P6 (Figure 3E and 3F). We therefore found evidence of either impaired RNA splicing (kindreds B, C, D, E, F, and G), or impaired protein production (kindreds A, B, C, D), or function (kindreds E and F) in all the kindreds tested. These data support the hypothesis that the intronic variants identified are pathogenic or likely pathogenic, in accordance with *American College of Medical Genetics and Genomics* (ACMG) criteria (14,21).

### Comparison of predicted and observed impacts on splicing

All BP and AG-gain variants were flagged as possibly pathogenic by several scores, including SpliceAI (19) and Pangolin (20), both of which provide an indication of the likely impact on the RNA. We, thus, compared the *in silico* predictions of these two scores (Table S2) with the results of a wet-laboratory RNA study for these variants (Figure 2, S1, S2 and Table 2). The observed impact of the *SH2D1A* BP variant in T-blasts from P2 (exon 2 skipping) was correctly predicted *in silico* by SpliceAI and Pangolin, both of which predicted an impact on the wild-type (WT) acceptor and donor splice sites of exon 2. For the *WAS* BP variant c.361-20T>G, both SpliceAI and Pangolin predicted the loss of the WT acceptor site of exon 4 but no impact on the donor site, consistent with the observed retention of intron 3 in the cells of P3. For the AG-gain variant c.2971-5C>A in *DOCK8*, both SpliceAI and Pangolin predicted the use of an alternative AG with an in-frame insertion of three nucleotides but a four-nucleotide insertion was actually observed in the T-blasts of P4. Interestingly, the intronic *DOCK8* revertant (c.2971-6C>G) was predicted by spliceAI to annihilate the AG-gain effect of the c.2971-5C>A. For the AG-gain variant of *STXBP2*, both SpliceAI and Pangolin predicted a major weakening of the WT acceptor site. However, discordant predictions were obtained for alternative AG sites. Indeed, SpliceAI predicted the creation of a new acceptor splice site at position c.1108-7 whereas Pangolin predicted the creation of a new acceptor splice site at position c.1108-32 (Figure S1A), which is what was actually observed in the T-blasts of P6. For the AG-gain variant of *UNC13D*, both SpliceAI and Pangolin predicted a weakening of the WT acceptor site and the creation of an acceptor site at c.2448-47, which was confirmed by the RNA study on T-blasts from P7. For the AG-gain variant of *NFKB1*, both SpliceAI and Pangolin predicted the creation of an acceptor splice site at c.572-31, whereas an acceptor splice site was actually created at c.572-32 in the T blasts of P7. Overall, the impact on RNA levels was corrected predicted for three of the six germline variants by SpliceAI and four of the six variants by Pangolin.

## DISCUSSION

We describe here seven new pathogenic or likely pathogenic intronic variants either disrupting BP, or leading to an AG-gain between the BP and the canonical acceptor site. The identification of these variants provided the various kindreds included in this study with a genetic diagnosis. BPHunter (17), AGain (18), splice AI (19), and Pangolin (20) were powerful tools after filtering based on the MAF of the variants. Nucleotide conservation-derived scores, such as phastCons (24) and CADD (23,32), can also be helpful, providing high scores for BP variants, but no interpretation of the likely effects of BP variants. There should be more systematic screening and investigation of intronic variants. Indeed, such variants can be called even with lower coverage study as suggested *in silico* by a 30X downsampling analysis (Table S3). The major advantage of IEI over other genetic diseases is that most IEI-related genes are expressed in peripheral blood cells and can be investigated through RNA studies and protein expression analysis. For genes that are poorly expressed in peripheral blood cells, or for variants that are subject to RNA nonsense mediated decay (NMD), treatment with an RNA inhibitor, such as emetine, can help to increase the number of reads (33). In clinical laboratory settings, such RNA studies are essential to confirm that the variant is pathogenic (PVS1 ACMG/AMP criterion) (14,15). They can also facilitate the investigation of variants at loci homologous to one or several pseudogenes (34-36) or screening for random monoallelic expression (37). The demonstration of an impairment of protein production or function by clinical flow cytometry also provides additional evidence of pathogenicity (38,39). A blind, hypothesis-free approach to RNA sequencing would also be worthwhile, but challenging, as it require many in-house controls and a dedicated bioinformatics pipeline, although such approaches have been shown to increase diagnostic yield slightly (40). In addition to the problem of possible variants of genes that are not expressed, such RNA studies are limited by the cell type-specificity of splicing (35). Our findings demonstrate the importance of the systematic screening and investigation of intronic variants in clinical laboratory settings for patients with suspected IEI but no genetic diagnosis.

## PATIENTS AND METHODS

### Case reports

#### Kindred A.

We investigated a 54-year-old patient (P1) with non-consanguineous Welsh parents living in France. His sister had undergone allogeneic bone marrow transplantation (BMT) for leukemia and is now being treated for colon cancer. The older brother of P1 was diagnosed with agammaglobulinemia on the basis of immunological tests during infancy. He was given lifelong intravenous immunoglobulin treatment, which unfortunately led to the transmission of hepatitis C virus (HCV), leading to cirrhosis and, ultimately, to the death of this individual. P1 has a history of chronic lung infections, including bronchial superinfections treated by antibiotics. Pulmonary function tests were normal. Given the clinical context and family history, immunological assessments were performed and showed: (i) hypogammaglobulinemia (IgG< 0.26 g/L (NR: 7 - 16 g/L), IgA < 0.05 g L (NR: 0.7 - 4 g/L) and IgM < 0.06g/L (NR: 0.4 – 2.3 g/L)), (ii) severe B-cell lymphopenia (CD19^+^ = 0/mm^3^; NR: 169–271/mm^3^) with normal T and NK cell counts and iii) an absence of antibody production (data not shown). P1 received immunoglobulin supplementation, initially intravenously, and then subcutaneously. After 15 years of treatment, his IgG levels are normal on supplementation and he remains free of pulmonary infections. Over the last two years, P1 has been complaining of undocumented chronic diarrhea not relieved by medication. Plans to provide IgA and IgM supplementation are currently being implemented.

#### Kindred B.

We investigated a single patient (P2) born in 2006 to non-consanguineous European parents living in France. The parents of the proband and his two sisters were healthy and had no remarkable medical history. P2 had a history of recurrent ear, nose, and throat (ENT) infections during infancy, including several acute middle ear infections treated by antibiotics that led to tympanoplasty. He also had two episodes of undocumented bronchitis and two episodes of scarlet fever. At the age of 16 years, he reported pain in the right tibia of several months’ duration, with no general signs apart from asthenia and weight loss. A magnetic resonance imaging (MRI) scan of the knee was performed, revealing a subperiosteal collection beneath the anterior tibial periosteum, with irregular intraosseous extension. A bone biopsy was performed and pathology examinations led to the diagnosis of a diffuse large B-cell lymphoma (DLBCL) of the germinal center (GC) subgroup. Immunological tests were performed. Blood cell counts and immunoglobulin levels were normal [IgG 7.3 g/L (normal range, NR: 7.0–16.0 g/L), IgA 0.82 g/L (NR: 0.8–3.0 g/L) and IgM 1.99 g/L (NR: 0.5–2g/L)] (Table S2) but isolated hypogammaglobulinemia due to IgG4 deficiency [IgG1 4.74 g/L (NR: 4.9–11.4 g/L), IgG2 1.3 g/L (NR: 1.5–6.4 g/L), IgG3 0.54 g/L (NR: 0.2–1.2 g/L) and IgG4 0.06 g/L (NR: 0.08-1.0 g/L)] was observed. Immunophenotyping showed that B and NK cell counts were normal but that the patient had severe T-cell lymphopenia (CD3^+^CD4^+^ 245/mm^3^, (NR: 500-1,500/mm^3^), CD3^+^CD8^+^ 312/mm^3^, (NR: 200-800/mm^3^), CD19^+^ 128/mm^3^, (NR: 100-800/mm^3^) and CD16^+^CD56^+^ 224/mm^3^, (NR: 50-400/mm^3^).

#### Kindred C.

P3 was born in 2015 to non-consanguineous parents. At the age of four months, he developed eczema. P3 has a history of upper and lower respiratory tract infections, and one episode of undocumented arthritis presumed to be bacterial. A CT-scan revealed bronchiectasis. This patient has also experienced episodes of epistaxis. Immunological tests revealed microthrombocytopenia and T-cell lymphopenia, with abnormally low percentages of naïve T cells. Levels of IgG, IgA, and IgM, and of toxoid tetanus were normal. The patient is currently treated by subcutaneous immunoglobulin supplementation, oral antibiotic prophylaxis (penicillin G and cotrimoxazole), and aerosolized salbutamol and steroids.

#### Kindred D.

P4 was born in 1985 to non-consanguineous parents. She has a history of several cutaneous and gynecologic HPV infections requiring frequent conizations and, more recently, urothelial cancer requiring surgery. She has also suffered from bronchiectasis with bronchial superinfections and *Pseudomonas aeruginosa* colonization. Immunophenotyping showed normal CD8^+^ T and NK cell counts but CD4^+^ T- and B-cell lymphopenia [CD3^+^CD4^+^ 206.32/mm^3^, (NR: 460-1230/mm^3^), CD3^+^CD8^+^ 614.26/mm^3^, (NR: 190-850/mm^3^), CD19^+^ 54.23/mm^3^, (NR: 92-420/mm^3^) and CD16^+^CD56^+^ 203.97/mm^3^, (NR: 89-362/mm^3^)]. Immunoglobulin levels were subnormal (IgG: 9.7 g/L (NR: 5.4–13.2 g/L), IgA 2.7 g/L (NR: 0.5–2.2 g/L) and IgM 0.42 g/L (NR: 0.53–1.62 g/L).

#### Kindred E.

We investigated a single patient (P5) born in 2006 to consanguineous Moroccan parents living in Morocco. P5 had four brothers. The eldest (I.1) died at the age of 11 years from Wilson's disease and a possible undocumented IEI. Another brother died at the age of five years from macrophagic activation syndrome (MAS), part of the spectrum of hemophagocytic lymphohistiocytosis (HLH). No genetic investigation was performed on either of these brothers. The parents of the proband and his other two brothers are healthy, and the patient had no other remarkable medical history. Since the age of two years, P5 has had a history of recurrent ENT infections, including pharyngitis, sore throats, and ear infections treated with antibiotics on an outpatient basis. At the age of four years, splenomegaly with progressive enlargement occurred. The patient also had three episodes of undocumented left basal lung disease during a single year that were treated with intravenous antibiotics, with a good clinical outcome. P5 is now suffering from lung disease, which is probably chronic due to repeated infections. He also has splenic involvement, with an enlarged spleen, possible hepatic involvement causing portal hypertension, and anemia due to iron deficiency. Immunological testing was performed when P5 was referred to our laboratory in France. Blood cell counts revealed cytopenia, including anemia, neutropenia, and thrombocytopenia [hemoglobin 6 g/dL (NR: 11.5-15.5 g/dL), neutrophils 0.740 (NR: 2-8 T/L) and platelets 86 T/L (NR: 150-450 T/L)]. Immunoglobulin levels were normal (IgG 11.6 g/L (NR: 5.4–13.2 g/L), IgA 0.77 g/L (NR: 0.5–2.2 g/L) and IgM 1.15 g/L (NR: 0.53–1.62 g/L)) (Table S2). In addition, immunophenotyping revealed normal T and NK cell counts but severe B-cell lymphopenia [CD3^+^CD4^+^ 1,183/mm^3^, (NR: 650-1,500/mm^3^), CD3^+^CD8^+^ 1,332/mm^3^, (NR: 370-1,100/mm^3^), CD19^+^ 35/mm^3^, (NR: 273-860/mm^3^) and CD16^+^CD56^+^ 220/mm^3^, (NR: 100-480/mm^3^)].

#### Kindred F.

P6 was born in 2003 to non-consanguineous parents. The parents of the proband and his brother were healthy and had no remarkable medical history. Since birth, P6 has suffered from hemophagocytic lymphohistiocytosis (HLH), treated by haploidentical hematopoietic stem cell transplantation with cells from his father at the age of six months. This procedure was successful and P6 remains in good health to this day.

#### Kindred G.

P7, born in 2004, has a history of upper respiratory tract infections since infancy. At the age of five years, he was hospitalized for meningitis due to enterovirus. At the age of 20 years, he displayed pneumonia with septicemia due to *Streptococcus pneumoniae,* which was successfully treated with antibiotics. Immunological testing was performed at the age of 17 years and revealed low immunoglobulin levels, normal counts of B cells, abnormally low percentages of switched memory B cells, and an excess of CD21^low^ cells. P7 was initially treated with cotrimoxazole, and then with immunoglobulin supplementation from the age of 20 years onwards. P7’s father, P8, born in 1966, also had a history of common variable immunodeficiency with low levels of immunoglobulin.

### Patient recruitment

Patients were recruited through our clinical laboratory, which is the reference laboratory in France for the investigation of IEI. All patients or their guardians provided written informed consent. The investigations described here were performed in France, in accordance with local regulations.

### High-throughput sequencing on genomic DNA

High-throughput sequencing was performed for a panel encompassing all known IEI genes, as previously described (10). The different version of the panel encompasses from 300 to 500 genes. DNA was extracted from EDTA-treated peripheral blood samples, either manually via the phenol-chloroform method, or with a Chemagic Prime instrument (Perkin Elmer). Genomic DNA libraries were generated from 2 μg DNA sheared with a Covaris S2 Ultrasonicator, with the SureSelectXT HS2 Library PrepKit [Agilent, Garches, France], on the Genomic Platform at the Imagine Institute, Paris. Capture was performed by hybridization, with 120 bp biotinylated complementary RNA baits designed with SureSelect SureDesign software (Agilent, H. sapiens, hg19, GRCh37, February 2009) to cover all exons and splicing junctions of the 450 genes implicated in IEIs. The targeted regions of interest were pulled out with magnetic streptavidin beads, amplified by PCR with indexing primers and sequenced on an Illumina HiSeq2500 HT system (paired-end sequencing, 2 x130 bases).

Data were analyzed at the University of Paris Cité/Imagine Institute Bioinformatics core facilities. Paired-end sequences were mapped onto the human reference genome (NCBI build37/hg19 version) with the Burrows-Wheeler Aligner. Downstream processing was performed with the Genome Analysis Toolkit (GATK), SAMtools (41) and Picard tools, according to documented best practice (https://software.broadinstitute.org/gatk/best-practices/). Variant calls were made with the GATK Unified Genotyper, based on the 72^nd^ version of the ENSEMBL database. Genome variants were defined with our in-house PolyDiag software for NGS, which filters out irrelevant and common polymorphisms on the basis of frequencies in public databases: the US National Center for Biotechnology Information Database of Single-Nucleotide Polymorphisms (SNPs) (dbSNP) (42), 1000 Genomes (43), Exome Variant Server (EVS, https:// evs.gs.washington.edu/EVS/), and the Genome Aggregation Database (gnomAD, https://gnomad.broadinstitute.org/) (44).

We evaluated CNVs (*i.e.* large duplications or deletions) for each individual, by determining the relative read count for each targeted region as the ratio of the read count for that region divided by the total absolute number of read counts for all the targeted regions. The ratio of the relative read count for a region in a given individual to the mean relative read count in other individuals from the same run gave the estimated CNV for the region concerned in the individual considered (method adapted from (45)). Homozygous deletion was suspected when this ratio was close to zero (no aligned reads). For the detection of monoallelic CNVs, a ratio below 0.7 was considered suggestive of heterozygous deletion, whereas a ratio above 1.3 was considered suggestive of heterozygous duplication.

### *In silico* screening of pathogenic variants

Variants were aligned with the hg37 or hg38 reference sequence and the following scores were determined: Again (https://hgidsoft.rockefeller.edu/AGAIN/; https://github.com/casanova-lab/AGAIN) (18), BPHunter (https://hgidsoft.rockefeller.edu/BPHunter/; https://github.com/casanova-lab/BPHunter) (17), spliceAI (https://spliceailookup.broadinstitute.org/; https://github.com/Illumina/SpliceAI); (19) and Pangolin (20) (https://spliceailookup.broadinstitute.org/; https://github.com/tkzeng/Pangolin), CADD v1.7 (https://cadd.gs.washington.edu/) (23), phastCons (http://compgen.cshl.edu/phast/) (24), and phyloP (http://compgen.cshl.edu/phast/) (25).

Possible pathogenic intronic variants were primarily screened using SpliceAI, Pangolin, AGain, and BPHunter. Regarding the cut-offs used, for spliceAI (and by homology for Pangolin), any absolute score ≥ 0.2 was considered as significantly elevated. This latter score is the permissive recommended cut-off by ACMG (14,15). For AGain and BPHunter, any score ≥ 3 was considered as significantly elevated. Results for other scores (CADD v1.7, phastCons, phyloP) are also provided for descriptive purposes but were not specifically used to screen for variants.

### Cell culture

PBMCs were isolated with Ficoll (#CMSMSL01-0U, Eurobio). T-blasts were generated from fresh or cryopreserved PBMCs with ImmunoCult-XF T-Cell Expansion Medium (#10981, StemCell) supplemented with ImmunoCult Human T-Cell Activators (#10970, StemCell) and interleukin-2.

### High-throughput sequencing on complementary DNA

Transcriptomic analysis was performed as previously described (33,46). Briefly, RNA was extracted from PBMCs or T-blasts (NucleoMag RNA kit for magnetic bead-based RNA purification, #744350, Macherey-Nagel). DNA was eliminated with DNaseI (#M03035, Ozyme). RNA was reverse-transcribed to generate cDNA (PrimeScript RT reagent kit with gDNA Eraser (Perfect Real Time),#RR047Q, Takara), the second strand was synthesized (Second-Strand cDNA Synthesis Kit, #A48570; Thermo Fisher Scientific), and the resulting cDNA was purified (AMPure XP Reagent, #A63881, Beckman Coulter). We then sequenced 10 to 25 ng purified double-stranded cDNA, using the previously described Agilent panel of IEI genes for capture. Data were then aligned as previously described (33,46). Sashimi plots were drawn with IGV, using splice junctions representing at least 5% of the mean coverage of the respective gene as the cut-off for minimal coverage.

### Flow cytometry protein expression

Intracellular flow cytometry was performed as follows. For BTK, whole blood cells were first subjected to extracellular staining with anti-CD14-Pacific blue (clone M5E2, #558121, BD) and anti-CD19 FITC (clone J3-119, #A07768, Beckman) antibodies, permeabilized with PhosphoFlow Lyse/fix buffer (#558049, Becton Dickinson) and Phosphoflow PermWash Buffer I (#557885, Becton Dickinson), and then incubated with monoclonal anti-BTK AF647 antibody (clone 53/BTK, #558528, Becton Dickinson) or the corresponding AF647 isotype (clone MOPC-173, #558053, Becton Dickinson). For SAP, T-blasts were first permeabilized with a 0.5% BSA 0.5% saponin buffer. They were then incubated with or without murine monoclonal antibody (clone 1C9, #H00004068-M01, Abnova), with an AlexaFluor488 goat anti-mouse secondary antibody for detection (Thermo Fisher Scientific). For WASp, T-blasts were permeabilized with Cytofix/Cytoperm (#554714, Beckton Dickinson) and incubated with monoclonal anti-WASp PE antibody (clone 5E5, custom reference, Beckton Dickinson) or the corresponding PE isotype (normal mouse IgG1, Beckton Dickinson). DOCK8 staining was performed as previously described (31). Briefly, whole blood cells were permeabilized with Cytofix/Cytoperm (#554714, Beckton Dickinson) and incubated with clone EPR12511 (#ab175208, Abcam) or left unstained. A goat AF647-coupled secondary antibody against rabbit IgG (polyclonal, #ab150083, Abcam) was used for detection.

### Degranulation or cytotoxicity assay

Degranulation assays were performed with Vγ9^+^Vδ2^+^ T cells as previously described (47). Cytotoxic activity was assessed as previously described (48). Briefly, PBMCs from patients, parents or normal controls were cultured with phytohemagglutinin (PHA, 1/700 dilution; Difco, Detroit, MI, USA) and IL-2 (20 IU/mL, Valbiotech, Paris, France) for 24 h. We then added IL-2 (40 IU/mL) and incubated the cells for six days. For the cytotoxic activity assay, the lysis of Fas-deficient L1210-3 target cells (10^4^ chromium^51^-loaded L1210 cells) as assessed in a standard four-hour release assay in the presence of monoclonal anti-CD3 antibody (OKT3; Ortoclone, Jansen, France). The effector/target ratio was calculated from the number of CD8^+^ T cells, as determined by flow cytometry.

## Supplementary Material

Supplementary Material **Figure S1 – IGV view of high-throughput sequencing of P4’s DNA extracted from whole blood (up) or T-blasts (below)**, in *DOCK8* at the junction of intron 24 and exon 25.
**Figure S2 – Sashimi plots of (A)** *DOCK8* with the skipping of exons 13 to 43 in T-blasts from P4 and **(B)** *NFKB1* exon 8 skipping in T-blasts from P7.
**Figure S3 – SpliceAI and Pangolin view** from https://spliceailookup.broadinstitute.org/ of AG-gain variant c.1108-9T>A in *STXBP2*.

Fig. S1 shows evidence for *DOCK8* revertant at genomic level in P4. Fig. S2 shows additional Sashimi plots for DOCK8 (P4) and NFKB1 (P7). Fig. S3 shows view for *STXBP2* in silico prediction by spliceAI and Pangolin. Table S1 shows a summary of the phenotype of the patients and their relatives. Table S2 shows the effects of the seven intronic variants predicted by SpliceAI and Pangolin. Table S3 show alignement and calling of the various variants in a 30X downsampling experiment.

## Figures and Tables

**Table 1 –:** Summary of the features of the intronic variants identified with the various associated scores

Kindred	Type of variant	Locus	Zygosity	gDNA position (hg38)	cDNA (MANE)	BPhunter score	AGain score	Absolute spliceAl max/ Pangolin max	CADD v1.7	phyloP	phastCons	MAF gnomAD v4.1.0
A	BP	*BTK*	Hemizygous	chrX-101354717-TG-T	c.1567-24del	4	NA	0.43/0.47	14.78	1.63	1	0
B	BP	*SH2D1A*	Hemizygous	chrX-124365739-A-G	c.138-22A>G	4	NA	0.81/0.64	20.2	5.24	1	0
C	BP	*WAS*	Hemizygous	chrX-48685714-T-G	c.361-20T>G	7	NA	0.54/0.36	7.994	3.08	0.99	0
D	AG-gain	*DOCK8* *	Heterozygous	chr9-396780-C-A	c.2971-5C>A	NA	5	0.99/0.83	20.83	1.77	0.03	0
E	AG-gain	*STXBP2*	Homozygous	chr19-7644605-T-A	c.1108-9T>A	NA	4	0.50/0.64	5.750	−0.61	0	0
F	AG-gain	*UNC13D* **	Heterozygous	chr17-75831397-G-T	c.2448-49C>A	NA	4	1/0.85	8.359	−0.28	0	0
G	AG-gain	*NFKB1*	Heterozygous	chr4-102578848-T-A	c.572-33T>A	NA	3	0.99/0.85	4.551	1.44	0.44	0

* In *trans* with a heterozygous deletion encompassing exons 13 to 43.

** In *trans* with the c.2927A>C p.H976P variant.

**Table 2 –:** Summary of the consequences of the various intronic variants detected at RNA level

Kindred	Cells and individuals studied	Gene and transcript references	Variant investigated	Consequences at RNA level
B	T-blasts from P2	*SH2D1A* (NM_002351.5)	c.138-22A>G	Skipping of exon 2, frameshift predicted
C	T-blasts from P3	*WAS* (NM_000377.3)	c.361-20T>G	Retention of intron 3, frameshift predicted
D	T-blasts from P4	*DOCK8* (NM_001290223.2)	c.2971-5C>A Δ13-43	Creation of a new acceptor splice site at −4, frameshift predicted (r.2970_2971insACAG, p.V991Gfs*11)
E	T-blasts from P5	*STXBP2* (NM_006949.4)	c.1108-9T>A	Creation of a new acceptor splice site at c.1108-32, frameshift predicted (r.1107_1108ins32, p.D370Rfs*6)
F	PBMCs from the mother of P6	*UNC13D* (NM_199242.3)	c.2448-49C>A	Creation of a new acceptor splice site at c.2448-47, frameshift predicted (r.2447_2448ins47, p.L817Hfs*18)
G	T-blasts from P7	*NFKB1* (NM_0039998.4)	c.572-33T>A	Creation of a new acceptor splice site at c.572-32, frameshift predicted (r.571_572ins32, p.D191Efs*17) Skipping of exon 8, frameshift predicted
