# Supplementary material for: Intronic branchpoint-to-acceptor variants underlying inborn errors of immunity
Source: J Hum Immun. 2025 Jul 17;1(3):e20250041. doi: 10.70962/jhi.20250041 (PMC12700597; doi:10.70962/jhi.20250041)
Supplement: Table S1 — shows the summary of clinical and basic immunological testing of index cases from the seven kindreds and their familial history. [file jhi_20250041_tables1.docx]

**Table S1 – Summary of clinical and basic immunological testing of index cases from the seven kindreds and their familial history**

|  | **Current age (y)** | **Sex** | **Clinical phenotype** | **Immunological phenotype** | **Familial history** |
| --- | --- | --- | --- | --- | --- |
| P1 | 56 | M | Intermittent chronic bronchopathy  Chronic diarrhea | Decrease in levels of IgG, IgA, and IgM  Absence of circulating B cells | II.1 hypogammaglobinemia |
| P2 | 19 | M | Recurrent ENT infections  Diffuse large-B-cell lymphoma | Hypogammaglobinemia  EBV-negative serology | None |
| P3 | 10 | M | Bleeding  Eczema | Microthrombocytopenia  CD8^+^ T-cell lymphopenia | None |
| P4 | 40 | F | Bronchiectasis  HPV-driven (cutaneous and gynecologic) infections | CD4^+^ T-cell lymphopenia  Decrease in DOCK8 expression (two peaks) | None |
| P5 | 8 | M | Hemophagocytic lymphohistiocytosis  Recurrent ENT infections | Excess of activated T cells | II.1 HLH  II.3 HLH |
| P6 | 22 | M | Hemophagocytic lymphohistiocytosis |  | None |
| P7 | 58 | M | Recurrent respiratory tract infections  Meningitis due to enterovirus | Low immunoglobulin levels |  |
| P8 | 20 | M | Recurrent respiratory tract infections | Low immunoglobulin levels |  |
