# Supplementary material for: Intronic branchpoint-to-acceptor variants underlying inborn errors of immunity
Source: J Hum Immun. 2025 Jul 17;1(3):e20250041. doi: 10.70962/jhi.20250041 (PMC12700597; doi:10.70962/jhi.20250041)
Supplement: Table S2 — shows the effects of the seven intronic variants predicted by SpliceAI and Pangolin. [file jhi_20250041_tables2.docx]

**Table S2 –** **Effects of the seven intronic variants predicted by SpliceAI and Pangolin**

| **Locus** | **cDNA**  **(MANE)** | **SpliceAI** | | | **Pangolin** | | |
| --- | --- | --- | --- | --- | --- | --- | --- |
|  |  | **Predicted WT acceptor loss** | **Other predicted**  **effects** | **Overall predicted**  **effect** | **Predicted WT acceptor loss** | **Other predicted**  **effects** | **Overall predicted**  **effect** |
| *BTK* | c.1567-24del | Yes, exon 16  (-0.3) | Acceptor gain at c.1567-138 (+0.47) | In-frame insertion  of 138 nucleotides at the beginning of exon 16 | Yes, exon 16  (-0.47) | Donor loss, exon 16  (-0.23) | Skipping of exon 16  (frameshift) |
| *SH2D1A* | c.138-22A>G | Yes, exon 2  (-0.77) | Donor lossn exon 2  (-0.81) | Skipping of exon 2  (frameshift) | Yes, exon 2  (-0.63) | Donor loss, exon 2  (-0.62) | Skipping of exon 2  (frameshift) |
| *WAS* | c.361-20T>G | Yes, exon 4  (-0.54) | None | Retention of intron 3  (frameshift) | Yes, exon 4  (-0.36) | None | Retention of intron 3  (frameshift) |
| *DOCK8* | c.2971-5C>A | No | Acceptor gain at c.2971-3C>A  (+0.99)* | In-frame insertion of 3 nucleotides at the start of exon 25* | Yes  (-0.25) | Acceptor gain at c.2971-3C>A  (+0.83) | In-frame insertion of 3 nucleotides at the start of exon 25 |
| *STXBP2* | c.1108-9T>A | Yes, exon 14  (-0.51) | Acceptor gain at c.1108-7T>A  (+0.46) | Frameshift insertion of 7 nucleotides at the start of exon 14 | Yes, exon 14  (-0.64) | Acceptor gain at c.1108-32T>A  (+0.4) | Frameshift insertion of 32 nucleotides at the start of exon 14 |
| *UNC13D* | c.2448-49C>A | Yes, exon 26  (-0.94) | Acceptor gain at c.2448-47  (+1) | Frameshift insertion of 47 nucleotides at the start of exon 26 | Yes, exon 26  (-0.59) | Acceptor gain at c.2448-47  (+0.85) | Frameshift insertion of 47 nucleotides at the start of exon 26 |
| *NFKB1* | c.572-33T>A | Yes, exon 26  (-0.72) | Acceptor gain at c.572-31  (+0.99) | Frameshift insertion of 31 nucleotides at the start of exon 8 | Yes, exon 26  (-0.67) | Acceptor gain at c.572-31  (+0.85) | Frameshift insertion of 31 nucleotides at the start of exon 8 |

*The *DOCK8* revertant (c.2971-6C>G) in P4 is predicted by spliceAI to annihilated the effect of the germline c.2971-5C>A variant.
