# Supplementary material for: Intronic branchpoint-to-acceptor variants underlying inborn errors of immunity
Source: J Hum Immun. 2025 Jul 17;1(3):e20250041. doi: 10.70962/jhi.20250041 (PMC12700597; doi:10.70962/jhi.20250041)
Supplement: Table S3 — shows the depth coverage of aligned and called intronic variants in the original and in 30X downsampling projects. [file jhi_20250041_tables3.docx]

**Table S3 –** Depth coverage of aligned and called intronic variants in the original and in 30X downsampling projects.

| **Locus** | **Zygosity** | **gDNA position**  **(hg38)** | **Variant (Wild-type/Mutated) depth coverage (X)** | |
| --- | --- | --- | --- | --- |
|  |  |  | **Original project** | **30X downsampling** |
| *BTK* | Hemizygous | X-101354717-TG-T | 0/130 | 0/7 |
| *SH2D1A* | Hemizygous | X-124365739-A-G | 0/275 | 0/21 |
| *WAS* | Hemizygous | X-48685714-T-G | 0/303 | 0/20 |
| *DOCK8* | Homozygous | 9-396780-C-A | 0/156 | 0/13 |
| *STXBP2* | Homozygous | 19-7644605-T-A | 0/323 | 0/30 |
| *UNC13D* | Heterozygous | 17-75831397-G-T | 363/348 | 24/20 |
| *NFKB1* | Heterozygous | 4-102578848-T-A | 140/136 | 7/4 |
