# Supplementary material for: Intronic branchpoint-to-acceptor variants underlying inborn errors of immunity
Source: J Hum Immun. Author manuscript; Available in PMC 2025 Dec 13. (PMC12700597; doi:10.70962/jhi.20250041)
Supplement: Supplementary file 1 [file NIHMS2116775-supplement-Supplementary_Material.pdf]

# Figure S1

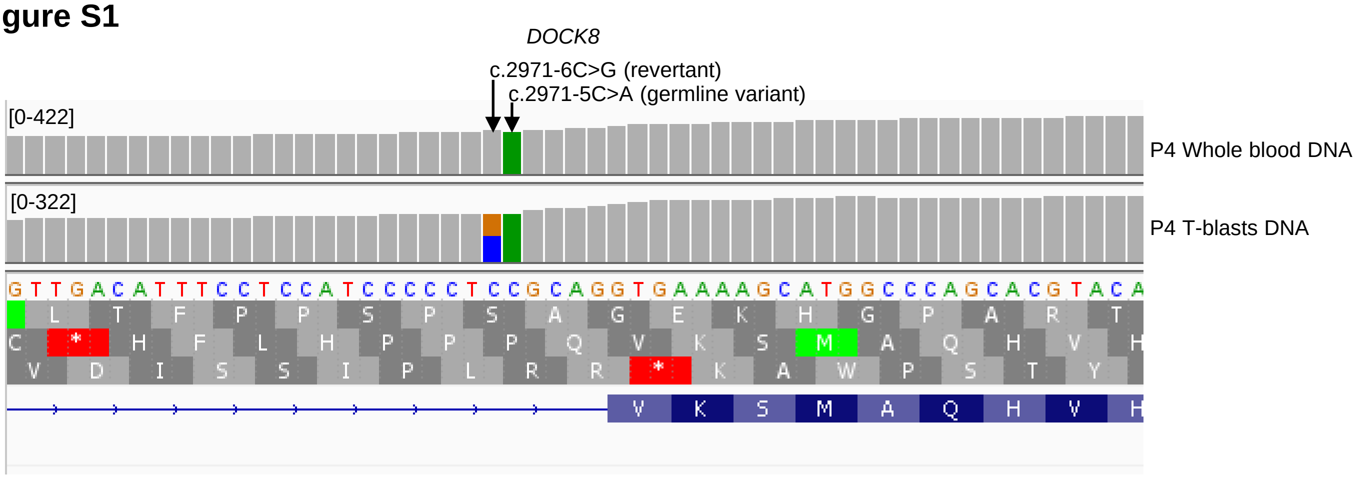

DOCK8
c.2971-6C>G (revertant)
c.2971-5C>A (germline variant)
[0-422]
P4 Whole blood DNA
[0-322]
P4 T-blasts DNA
G T T G A C A T T T C C T C C A T C C C C C T C C G C A G G T G A A A A G C A T G G C C C A G C A C G T A C A
L T F P P S P S A G E K H G P A R T
C * H F L H P P P Q V K S M A Q H V H
V D I S S I P L R R * K A W P S T Y
V K S M A Q H V H

# Figure S2

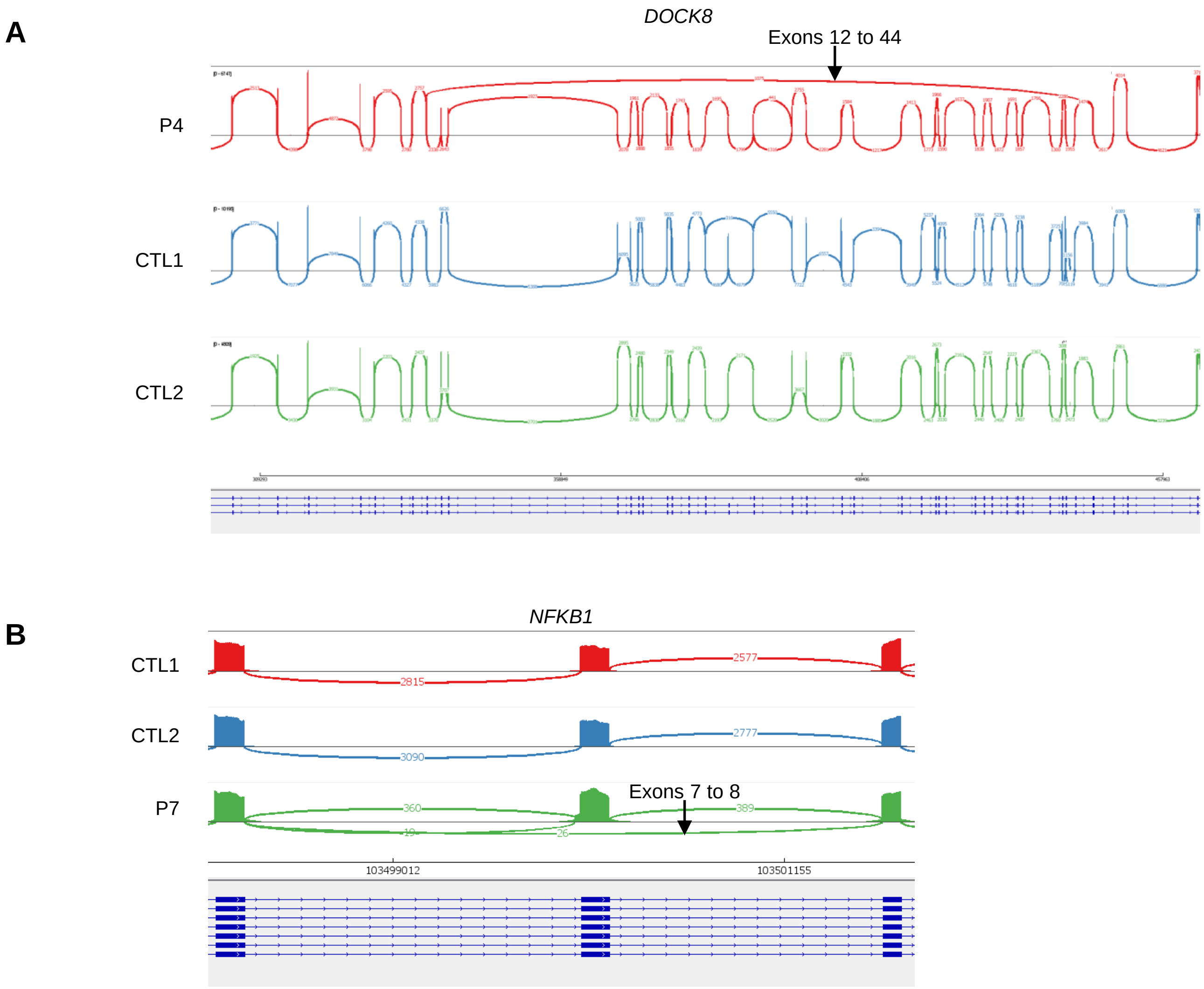
A
DOCK8
Exons 12 to 44
P4
CTL1
CTL2
B
NFKB1
CTL1
2815
2577
CTL2
3090
2777
P7
Exons 7 to 8
360
389
19
26
103499012
103501155

# Figure S3

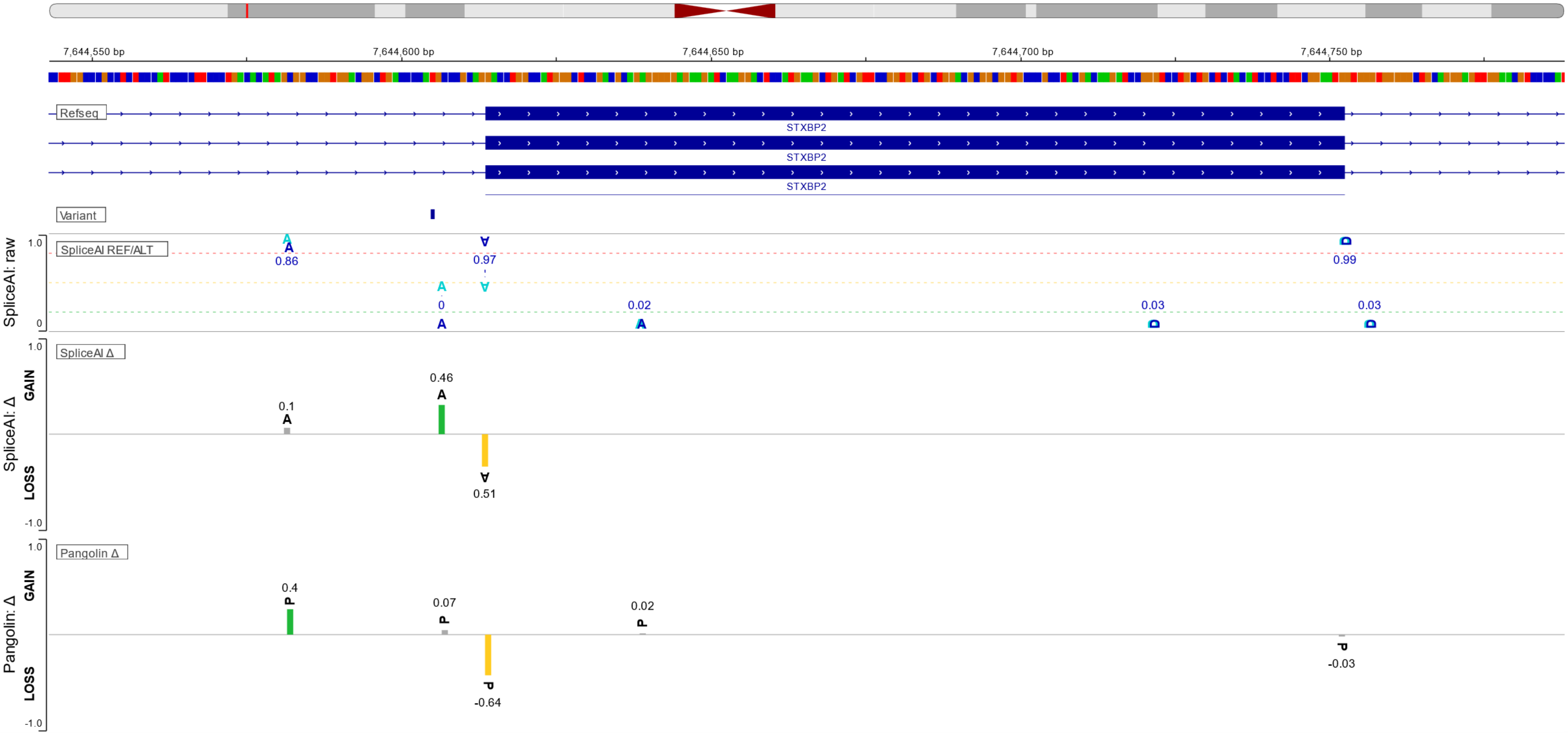

7,644,550 bp
7,644,600 bp
7,644,650 bp
7,644,700 bp
7,644,750 bp
Refseq
STXBP2
STXBP2
STXBP2
Variant
SpliceAI: raw
SpliceAI REF/ALT
1.0
0
0.86
0.97
0.99
0
0.02
0.03
0.03
SpliceAI: Δ
SpliceAI Δ
GAIN
LOSS
1.0
-1.0
0.46
0.1
0.51
Pangolin: Δ
Pangolin Δ
GAIN
LOSS
1.0
-1.0
0.4
0.07
0.02
-0.03
-0.64

## Table S1 – Summary of clinical and basic immunological testing of index cases from the seven kindreds and their familial history

| | Current age (y) | Sex | Clinical phenotype | Immunological phenotype | Familial history |
|---|---|---|---|---|---|
| P1 | 56 | M | Intermittent chronic bronchopathy<br>Chronic diarrhea | Decrease in levels of IgG, IgA, and IgM<br>Absence of circulating B cells | II.1 hypogammaglobinemia |
| P2 | 19 | M | Recurrent ENT infections<br>Diffuse large-B-cell lymphoma | Hypogammaglobinemia<br>EBV-negative serology | None |
| P3 | 10 | M | Bleeding<br>Eczema | Microthrombocytopenia<br>$CD8^+$ T-cell lymphopenia | None |
| P4 | 40 | F | Bronchiectasis<br>HPV-driven (cutaneous and gynecologic) infections | $CD4^+$ T-cell lymphopenia<br>Decrease in DOCK8 expression (two peaks) | None |
| P5 | 8 | M | Hemophagocytic lymphohistiocytosis<br>Recurrent ENT infections | Excess of activated T cells | II.1 HLH<br>II.3 HLH |
| P6 | 22 | M | Hemophagocytic lymphohistiocytosis | | None |
| P7 | 58 | M | Recurrent respiratory tract infections<br>Meningitis due to enterovirus | Low immunoglobulin levels | |
| P8 | 20 | M | Recurrent respiratory tract infections | Low immunoglobulin levels | |

## Table S2 – Effects of the seven intronic variants predicted by SpliceAI and Pangolin

| Locus | cDNA (MANE) | SpliceAI | | | Pangolin | | |
|---|---|---|---|---|---|---|---|
| | | Predicted WT acceptor loss | Other predicted effects | Overall predicted effect | Predicted WT acceptor loss | Other predicted effects | Overall predicted effect |
| *BTK* | c.1567-24del | Yes, exon 16 (-0.3) | Acceptor gain at c.1567-138 (+0.47) | In-frame insertion of 138 nucleotides at the beginning of exon 16 | Yes, exon 16 (-0.47) | Donor loss, exon 16 (-0.23) | Skipping of exon 16 (frameshift) |
| *SH2D1A* | c.138-22A>G | Yes, exon 2 (-0.77) | Donor lossn exon 2 (-0.81) | Skipping of exon 2 (frameshift) | Yes, exon 2 (-0.63) | Donor loss, exon 2 (-0.62) | Skipping of exon 2 (frameshift) |
| *WAS* | c.361-20T>G | Yes, exon 4 (-0.54) | None | Retention of intron 3 (frameshift) | Yes, exon 4 (-0.36) | None | Retention of intron 3 (frameshift) |
| *DOCK8* | c.2971-5C>A | No | Acceptor gain at c.2971-3C>A (+0.99)* | In-frame insertion of 3 nucleotides at the start of exon 25* | Yes (-0.25) | Acceptor gain at c.2971-3C>A (+0.83) | In-frame insertion of 3 nucleotides at the start of exon 25 |
| *STXBP2* | c.1108-9T>A | Yes, exon 14 (-0.51) | Acceptor gain at c.1108-7T>A (+0.46) | Frameshift insertion of 7 nucleotides at the start of exon 14 | Yes, exon 14 (-0.64) | Acceptor gain at c.1108-32T>A (+0.4) | Frameshift insertion of 32 nucleotides at the start of exon 14 |
| *UNC13D* | c.2448-49C>A | Yes, exon 26 (-0.94) | Acceptor gain at c.2448-47 (+1) | Frameshift insertion of 47 nucleotides at the start of exon 26 | Yes, exon 26 (-0.59) | Acceptor gain at c.2448-47 (+0.85) | Frameshift insertion of 47 nucleotides at the start of exon 26 |
| *NFKB1* | c.572-33T>A | Yes, exon 26 (-0.72) | Acceptor gain at c.572-31 (+0.99) | Frameshift insertion of 31 nucleotides at the start of exon 8 | Yes, exon 26 (-0.67) | Acceptor gain at c.572-31 (+0.85) | Frameshift insertion of 31 nucleotides at the start of exon 8 |

*The *DOCK8* revertant (c.2971-6C>G) in P4 is predicted by spliceAI to annihilated the effect of the germline c.2971-5C>A variant.

**Table S3** – Depth coverage of aligned and called intronic variants in the original and in 30X downsampling projects.

| Locus | Zygosity | gDNA position (hg38) | Variant (Wild-type/Mutated) depth coverage (X) | |
|---|---|---|---|---|
| | | | Original project | 30X downsampling |
| *BTK* | Hemizygous | X-101354717-TG-T | 0/130 | 0/7 |
| *SH2D1A* | Hemizygous | X-124365739-A-G | 0/275 | 0/21 |
| *WAS* | Hemizygous | X-48685714-T-G | 0/303 | 0/20 |
| *DOCK8* | Homozygous | 9-396780-C-A | 0/156 | 0/13 |
| *STXBP2* | Homozygous | 19-7644605-T-A | 0/323 | 0/30 |
| *UNC13D* | Heterozygous | 17-75831397-G-T | 363/348 | 24/20 |
| *NFKB1* | Heterozygous | 4-102578848-T-A | 140/136 | 7/4 |
